# Book Notices

**Published:** 1856-03

**Authors:** 


### BOOK NOTICES.

*Fourth Annual Review of the Commerce, Manufactures, and the Public and Private Improvement of Chicago, for the year* 1855; *with a full statements of her system of Rail Roads, and a general synopsis of the business of the City.* Compiled from several Articles in the Daily Democratic Press.

The above is the title of a neatly printed pamphlet of 80 pages, and though having no relation to *Medical* matters, we quote it for the purpose of informing such of our readers, as may desire information concerning any of the topics named in the above title, that they can obtain it by applying for a copy of the pamphlet at the Democratic Press Office, 45 Clark Street, and paying the small sum of *twenty-five cents.*

*The Half-Yearly Abstract of the Medical Sciences.* Edited by W. H. Ranking, M. D. and C. B. Radcliffe, M. D., and Re-published by Lindsay & Blackiston: Philadelphia, 1856.

This useful Abstract of the Medical Periodical Literature, for the last half of 1855, has appeared with its usual punctuality, and is worthy of the patronage of the profession. Price $2,00 per annum.

---

*The British and Foreign Medico-Chirurgical Review, or Quarterly Journal of Practical Medicine and Surgery, No.* 33. Re-published by S. S. & W. Wood, No. 261 Pearl Street, New York.

The January number of this able foreign quarterly is no less interesting than its predecessors. We notice in the list of Books reviewed in this number, the titles of three American Works, viz.:

"*A Practical Treatise on Foreign Bodies in the Air Passages.* By S. D. Gross, M.D. and Prof., of Louisville, Ky."

"*Clinical Lectures on Diseases of Women and Children.* By Gunning S. Bedford, A.M., M.D., &c., of New York;" and

"*Letters to a Young Physician just entering upon Practice.* By James Jackson, M.D., LL.D., &c., &c., of Boston."

The first and the last of the works here mentioned, meet the unqualified approbation of their reviewers; while the style of the second meets with some brief, but well merited, criticisms.

---

*Annual Report of the Surgeons of the New York Opthalmic Hospital, for the Year* 1855.

This is a pamphlet of 16 pages, containing the rules and regulations of the Institution named, which is located at No. 6 Stuyvesant Street, New York City; and under the professional charge of Mark Stephenson, M.D., and John P. Garrish, M.D., as surgeons.

The whole number of patients reported as having been registered on the books of the Hospital during the year 1855, was 1202. Of these, 602 were discharged cured; 323 relieved; 74 incurable; 18 declined treatment; 15 were removed to Bellevue Hospital; 173 discharged without result of treatment being known; and 77 remained in the Institution at date of report.

*Proceedings of the American Pharmaceutical Association, at the Fourth Annual Meeting, held in New York, September 11th, 12th and 13th.* Published by direction of the Association.

The above is the title of a neatly printed pamphlet of 40 pages, containing an abstract of the proceedings of the fourth annual meeting of the above named association; the constitution, officers, and list of members of the association; with an appendix, containing, 1st. A report on Standards for Drugs; 2d. An essay on the Growth of Wines in the West, and on Catawba Brandy and Tartar, by E. S. Wayne, of Cincinnati; 3d. A report on the subject of the Home Adulterations of Drugs; 4th. A report on the strength of Commercial Muriatic and Nitric Acids and Alcohol; 5th. Report of the Executive Committee; and 6th. A report on the inquiry whether any and what amendments are required to the law regulating the Importation of Drugs and Medicines, &c. These are all short papers, but containing matter of interest both to the profession and the community. The third report above-named we copy entire, as follows, viz:

"The subject of home adulterations of drugs naturally attracted the attention of the community, and especially of pharmaciens and physicians, in connection with that of the foreign, to which we have applied so stringent a law.

No doubt the sophistication of drugs is as well understood in this country as on the other side of the Atlantic, and that if we could apply a remedy as general in its application, we should detect an amount equally astonishing. This is one of the arguments used by the opponents of the drug law, that medicines can be as readily adulterated here as abroad; but we contend that this is no argument against shutting out foreign adulteration, and we hope some of these days to put a stop to the evil at home. The precise method of doing this is not yet apparent, neither is it within the scope of the duties of this Committee to suggest a remedy. One of the results of the different reports from time to time upon this subject, will be to call the attention of the community to the subject, and create a public sentiment that shall demand purer and better medicines when needed, thus drawing the necessary discrimination between the qualities of them when offered either in packages or at retail.

The Committee do not design, at present, a full report, as there are still under their observation, and that of others who have aided them in this matter, such articles as are usually met with. Some are of more, some of less importance, all however, sufficiently so, we think, to merit attention and remark. They are mostly articles that have been found on sale in the interior towns and cities, purchased at the cities East, where most of the wholesaleing is done. A few instances may be noticed:—

*Balsam Peru* has been met with, possessing none of the characteristics of genuine balsam except in color and consistency, and upon analysis affording no cinnamic acid.

*Pulv. Capsicum.*—The sample examined had a brick dust color, little pungency, and filled with yellow specks and strong odor of *tumeric.* It was a mixture of tumeric and American capsicum, and, of course almost inert.

*Castor* is found with the follicles filled with saw dust to half the weight of the castor.

*Opium.*—Since the circular of the Secretary of the Treasury fixing a per centage of morphia for this drug, a more uniform quality has been found in market; but a great many samples have been observed the past season with foreign substances, most commonly lead, inserted in the lumps, in some instances equal to 20 per cent. of the weight of the mass. We are of the opinion that this was done abroad, and probably at the port whence shipped. The different examiners should seek to detect this fraud before passing it.

*Musk* in pod has been observed loaded in the same way, to the amount of 20 grains in a single pod.

*The Essential Oils* are largely adulterated in this country.

*Oil of Peppermint* sometimes contains 50 per cent. of alcohol. *Oil of Rosemary* is adulterated largely with turpentine, and in short, the whole class are shamefully sophisticated.

*Otto of Rose* in the same class.

*Cream of Tartar*, adulterated with carbonate of lime, some samples to the extent of 33 per cent., others in less proportion. Sul. potash is also used for this purpose, and alum largely. Of six specimens examined by a gentleman of New York City, purchased at various shops, but *one* was found pure, some of them being adulterated 30 per cent. The same gentleman says, in reply to our enquiries, that from twenty-two specimens or samples of essential oils, fourteen were found to contain turpentine and other impurities. The same gentleman reports samples of powdered opium adulterated 50 per cent.

*Cod Liver Oil.*—All kinds of fish oil may be found neatly bottled and carefully labelled as the genuine article.

*Sulphate of Quinine.*—Samples have been detected with the old adulteration of mannite, and one gentleman reports quinine mixed up with fine picked raw cotton, adding to the bulk so as to fill the vial without using the requisite quantity of this valuable chemical.

Ipecacuanha in powder and Jalap in powder, each mixed with spurious matter, and English rhubarb in powder, put up for fine powdered Turkey, are not uncommon in all the markets.

Of crude materials, *Nitre*, or Saltpetre is one of the most commonly sophisticated, being adulterated with common salt and nitrate of soda largely.

These are some of the reports made to us, all from reliable sources.

The Committee have endeavored to establish points of observation in different sections of the United States, and as far as possible to obtain the names of houses from whom these various sophistications have been obtained. Such information they deem it best to withhold from publication at present, lest they might do injustice to parties ignorantly sending out such drugs; but they also intend, from time to time, to compare notes, and when satisfied of continued practices of this kind, will report such names to the Association.

In the meantime, they cannot too strongly urge retail apothecaries especially, to be cautious of whom and what quality of medicines they purchase. It is to the dispensing apothecary medical men and the community look for such medicines as are pure, not only 'good of their kind,' but of the best kind.

C. B. Guthrie,
Geo. D. Goggeshall,
E. S. Wayne,
A. J. Matthews."

The following Prizes are also offered by the Association:—

1*st.—Twenty-three volumes of the American Journal of Pharmacy.*

For the best Essay which shall develope the commercial history of all drugs indigenous to the United States, as Senega, Spigelia, Serpentaria, &c., as regards the manner and places of their collection and preparation for the supply of commerce, the amount annually collected, and the channels through which they enter general commerce.

2*nd.—Six volumes of Gmelin's Hand Book of Chemistry.*

For the best Essay on any question relating specially to Pharmacy.

COMMITTEE OF JUDGES.

CHARLES ELLIS. WILLIAM PROCTOR, Jr.

All Essays contributed for the Prizes must be delivered free of charge to CHARLES ELLIS, Philadelphia, on or before the second Tuesday in August, 1856.

